# Paramedical staffs knowledge and attitudes towards antimicrobial resistance in Dire Dawa, Ethiopia: a cross sectional study

**DOI:** 10.1186/s12941-017-0241-x

**Published:** 2017-09-19

**Authors:** Belay Tafa, Adugna Endale, Desalegn Bekele

**Affiliations:** 1 School of Medicine, College of Medicine and Health Sciences, Dire Dawa University, Dire Dawa, Ethiopia; 2 Department of Medical Laboratory Sciences, College of Medicine and Health Sciences, Ambo University, Ambo, Ethiopia

**Keywords:** Paramedical staffs, Antimicrobial resistance, Knowledge, Attitudes

## Abstract

**Background:**

The continuing emergence, development and spread of pathogenic organisms that are resistant to antimicrobials are a cause of increasing concern. The control of antimicrobial resistance requires knowledge of factors causing antimicrobial resistance, good attitudes towards the intervention strategies as well as changes in antibiotic prescribing behavior of health workers. Hence, this study was aimed to assess paramedical staffs’ knowledge and attitudes towards antimicrobial resistance and their antibiotics prescription practices in Dire Dawa, Ethiopia.

**Methods:**

A cross-sectional survey was conducted among paramedical staffs working in hospitals and health centers. A total of 218 paramedical staffs were participated and a self-administered questionnaire was used to collect data. Data was analyzed using SPSS version 20. Chi square/Fisher’s exact tests were used for comparison of data and a p value of less than 0.05 was considered statistically significant.

**Results:**

Out of the total, 137 (62.8%) of paramedical staffs had good knowledge on the factors causing antimicrobial resistance. The most common causes of antimicrobial resistance reported were patients’ poor adherence (96.5%), self prescription (95%), and empiric choice of antibiotics (94.5%). In general, more than 80% of the respondents had positive attitudes towards the antimicrobials resistance intervention strategies. Relatively less proportion of participants recognized that antimicrobial resistance as a problem in their local institutions. The most perceived driving forces for unnecessary antibiotics prescriptions were treatment failure (67.7%) and patient push (53.3%). The majority, 76.9% of the prescribers mentioned that standard treatment guidelines were available in their institutions though only 15.7% of them reported referring the guidelines on the daily basis. Among the prescribers, 85.8% never attended formal trainings on antibiotics prescriptions.

**Conclusions:**

As this study generated important information on knowledge and attitudes of paramedical staffs about antimicrobial resistance, it identified areas of misconceptions and specific groups to be targeted for educational interventions regarding antimicrobial resistance. It is, therefore, suggested that a well-planned, organized and structured training programs should be undertaken to improve the appropriate use of antibiotics.

## Background

Antimicrobial resistance is a form of drug resistance whereby some (or, less commonly, all) sub-populations of a microorganism, usually a bacterial species, are able to survive after exposure to one or more antimicrobial agents [1].

Antibiotic resistance is a serious and growing phenomenon in contemporary medicine and has emerged as one of the pre-eminent public health concerns of the twenty first century, in particular as it pertains to pathogenic organisms. It is happening in every region of the world and has the potential to affect anyone, of any age, in any country, being a major threat to public health [2].

The continuing emergence, development and spread of pathogenic organisms that are resistant to antimicrobials are a cause of increasing concern. People infected with drug-resistant organisms are more likely to have longer and more expensive hospital stays, and may be more likely to die as a result of the infection. When the drug of choice for treating their infection doesn’t work, they require treatment with second- or third-choice drugs that may be less effective, more toxic, and more expensive or may be locally unavailable. This means that patients with an antimicrobial-resistant infection may suffer more and pay more for treatment [3].

Antibiotic resistance is causing not only increased morbidity and mortality but also a high economic burden, adding a load to health systems in low income countries which are already struggling with chronic underfunding and weak institutional structures [4].

In Ethiopia, the effectiveness of currently available antimicrobial drugs is decreasing due to the increasing number of resistant strains causing infections so that available therapeutic options for such organisms are severely limited. The overall multidrug resistance among bacterial isolates was 59–79% [5, 6]. Such widespread resistance to antimicrobial classes is something serious because a few treatment options remain for patients with bacterial infections.

In healthcare settings, medical equipments are common reservoirs for pathogenic bacterial strains being a potential vehicle in the transmission of multidrug resistant infections between patients and Healthcare Workers [7]. In eastern Ethiopia, a high level of antimicrobial resistance was detected for pathogenic bacteria; even showing complete resistance for commonly used antimicrobial agents [8].

The main factors that drive antimicrobial resistance in low income countries are irrational drug use such as over-prescription and unnecessary prescription of antibiotics (such as for viral infections), incomplete treatments and self-medication as well as insufficient infection control measures to prevent spread of resistant bacteria both in the community and the hospital [9, 10]. Poor hand hygiene by hospital staff has been associated with the spread of resistant organisms [11].

To our knowledge, only one study was undertaken to assess knowledge and beliefs about antimicrobial resistance among physicians and nurses in Ethiopia [12]; however the knowledge, attitude and practices of other paramedical staffs have not yet addressed. In general, there are a limited number of literatures on the issue in Ethiopia. Hence, this study assessed paramedical staffs’ knowledge and attitudes towards antimicrobial resistance and their antibiotics prescription practices.

## Methods

### Study design, period and setting

A descriptive cross sectional study was conducted from October to November 2015 in Dire Dawa Administration which is located in the eastern part of Ethiopia 515 km from Addis Ababa, the capital of the country. The total area of the administration is 128,802 hectare and the administration shares common boundaries with Ethiopian Somali Regional States in the West, North and East, and with Oromia Regional State in the South. In Dire Dawa Administration, there are 7 hospitals (5 private and 2 governmental) and 15 health centers serving the community.

### Source population

All paramedical staffs holding bachelor degree (Nurses, Health Officers, Midwives, Medical Laboratory Technologists, and Pharmacists) working in hospitals (private and governmental) and health centers in Dire Dawa Administration were source population for the study.

### Study population and sample size

A total of 218 paramedical staffs (41 Health Officers, 96 BSc Nurses, 31 Pharmacists, 21 Midwives, and 29 Medical Laboratory Technologists) who volunteered were included in this study.

### Data collection instruments

The data was collected from the participants using a self-administered pretested questionnaire. The questionnaire contained 6 parts and a total of 46 questions. In the first part, participants were asked to provide information about their socio-demographic data and professional profiles (10 items). The second part consisted of questions that assessed their knowledge regarding factors causing antimicrobial resistance (14 items). The participants knowledge was assessed by using a 4-point Likert scale, and the likert scale was dichotomized as correct answer (strongly agree and agree responses) and incorrect answer (disagree and strongly disagree responses).

The third part evaluated health professionals’ attitudes regarding intervention measures of antimicrobial resistance (9 items). The fourth part (3 items) and the fifth part (4 items) of the questionnaire addressed health professionals’ attitudes regarding the burden of antimicrobial resistance and causes of unnecessary antibiotic prescription conditions, respectively. These questions were analyzed by using a 5-point Likert scale, whose responses were “strongly agree, agree, disagree, strongly disagree, and don’t know”.

The sixth part (7 items) assessed health professionals’ antibiotic prescription practices and different response styles were used for assessing the prescribers practices. The 46 questions included in the survey were prepared by a thorough literature search of the published studies that are relevant to our survey. The questionnaire was prepared in English and used for the survey.

### Data quality assurance

The quality of the data was assured through pretesting of the questionnaire, training of data collectors and supervisors, close supervision of the data collectors and proper handling of the data. It was monitored frequently both in the field and during data entry that all questionnaires were checked for completeness and consistency at the end of each day. Data entry was carefully done by a trained and experienced data clerk.

### Statistical analysis

The entire data was entered into a computer and analyzed using SPSS version 20.0. Frequency and percentages among descriptive statistics were used to describe the data. Chi square/Fisher’s exact test was used to assess the differences among variables. p value of < 0.05 was taken as statistical significant. The response alternatives for knowledge items were dichotomized and each question was assigned one mark for correct response and zero for incorrect response. Average score was considered as a cut off value and those scored equal to or greater were said to have good knowledge while those scored below the average were said to have poor knowledge. The questions on attitudes and some of prescription practices used Likert-style responses and frequencies and percentages were used to describe the results.

## Results

### Socio-demographic data and professional profiles

Socio-demographic data and professional profiles of the participants were described in Table 1. A total of 218 paramedical staffs were participated in the study. Of the total, 111 (50.9%) were from health centers and 107 (49.1%) were from hospitals. Of the participants, 166 (76.1%) were from urban health facilities while the rest 52 (23.9%) were from rural facilities. The majority of the study subjects were male accounting 67% (146 out of 218). Regarding the age of the participants, the majority 181 (83%) were in the interval of 21–30 years and the rest 21 (13.3%) and 8 (3.7%) individuals were in the age category of 31–39 and greater or equal to 40 years, respectively.

**Table 1:** Socio-demographic data and professional profiles of study subjects, Dire Dawa, Ethiopia, October to November 2015

Variables	Categories	Frequency	%
Sex	Male	146	67.0
	Female	72	33.0
Age group	20–29	181	83.0
	30–39	29	13.3
	≥ 40	8	3.7
Facility currently working	Hospital	107	49.1
	Health center	111	50.9
Location of the institution	Urban	166	76.1
	Rural	52	23.9
Ownership of the institution	Government only	184	84.4
	Private only	28	12.8
	Both government and private	6	2.8
Profession	BSc nurse	96	44.0
	Health officer	41	18.8
	BSc Midwifery	21	9.6
	Medical Lab Technologist	29	13.3
	Pharmacist	31	14.2
Work experience (years)	1–5	153	70.2
	6–10	42	19.3
	11–15	17	7.8
	≥ 16	6	2.8
Have you attended trainings on antimicrobial resistance?	Yes	21	9.6
	No	197	90.4
Have you ever used antimicrobial sensitivity test result for treating patients?	Yes	7	3.2
	No	211	96.8
Do you get up to date information on antimicrobial resistance?	Yes	101	46.3
	No	117	53.7
Source of information (n = 101)	Books and school course	46	45.5
	Internet and medical journals	35	34.7
	Trainings and discussion with colleagues	20	19.8

Of the participants by profession, the majority were nurses, 96 (44%) followed by Health Officers, 41 (18.8%). Of the respondents, 184 (84.4%) were working at government institutions and only 28 (12.8%) were working at private facilities while a few, 6 (2.8%) of the participants were at both private and government facilities.

Regarding work experience, 153 (70.2%) of the study participants had working experiences of 1–5 years and 42 (19.3%), 17 (7.8%), and 6 (2.8%) had experiences of 6–10, 11–15, and greater or equal to 16 years, respectively.

Of the study participants, the majority 197 (90.4%) had not attended any training on antimicrobial resistance. The greater proportion, 96.8% (211/218) of the study subjects had not used antimicrobial sensitivity test results for treating the patients.

Of the subjects, 101 (46.3%) got up to date information on antimicrobial resistance from different sources but 117 (53.7%) did not respond getting of the information. From those getting up to date information about antimicrobial resistance (n = 101), 46 (45.5%) reported books and school courses, 35 (34.7%) internet and journals and 20 (19.9%) trainings and discussions with colleagues as their sources.

### Knowledge regarding factors causing antimicrobial resistance

Based on the respondents survey results of knowledge concerning the causes of antimicrobial resistance, the common causes for antimicrobial resistance were patient poor adherence, 210 (96.5%), self prescription, 207 (95%), and empiric choice of antibiotics, 206 (94.5%). Of the respondents, 202 (92.7%) considered that each widespread/overuse of antibiotics, inappropriate duration of antibiotic courses and poor infection control contributed for antimicrobial resistance. Prescribers poor awareness, sub-standard quality of antibiotics and microbes mutation promote antimicrobial resistance as responded by 196 (89.9%), 195 (89.4%) and 195 (89.4%) of the participants, respectively. Respectively, 136 (62.4%) and 127 (58.3%) of the participants answered that the use of broad spectrum antibiotics and promotion by pharmaceutical representatives for antibiotics promotes antimicrobial resistance (Table 2).

**Table 2:** Percentage of respondents giving correct responses to knowledge statements, Dire Dawa, Ethiopia, October to November 2015

Statements	Correct response		Incorrect response	
	Number	%	Number	%
Widespread or over use of antibiotics promotes antimicrobial resistance	202	92.7	16	7.3
Inappropriate empiric choices promote antimicrobial resistance	206	94.5	12	5.5
Inappropriate duration of antibiotics course promotes antimicrobial resistance	202	92.7	16	7.3
Use of broad spectrum antibiotics promotes antimicrobial resistance	136	62.4	82	37.6
Poor access to local antibiogram data promotes antimicrobial resistance	168	77.1	50	22.9
Microbe mutations cause antimicrobial resistance	195	89.4	23	10.6
Promotion by pharmaceutical representatives to antibiotics promotes antimicrobial resistance	127	58.3	91	41.7
Patient demands and expectations promote antimicrobial resistance	157	72	61	28
Prescribers’ poor awareness promotes antimicrobial resistance	196	89.9	22	10.1
Self-prescription by patients promotes antimicrobial resistance	207	95	11	5
Poor hand-washing in healthcare settings spread antimicrobial resistance	157	72	61	28
Poor infection control in hospitals spread antimicrobial resistance	202	92.7	16	7.3
Patient poor adherence promotes antimicrobial resistance	210	96.3	8	3.7
Sub-standard quality of antibiotics promotes antimicrobial resistance	195	89.4	23	10.6

A total of 14 questions were asked regarding factors causing antimicrobial resistance (Table 2), and the average response score was found to be 12. The majority of the participants, 137 (62.8%) responded equal to or more than the average value (considered as having good knowledge) while 81 (37.2%) of the participant scored less than the average score (considered as having poor knowledge). Statistically significant differences in the level of knowledge were noted between sex (p = 0.044), age groups (p = 0.004), ownership of the institution (p = 0.004), location of the institution (p = 0.018) and profession of the participants (p = 0.004) (Table 3).

**Table 3:** Association of socio-demographic characteristics and professional profiles with level of knowledge, Dire Dawa, Ethiopia, October to November 2015

Variables	Categories	Level of knowledge		p value
		Good number (%)	Poor number (%)	
Sex	Male	99 (67.8)	47 (32.2)	0.044
	Female	38 (52.8)	34 (47.2)	
Age group	20–29	109 (60.2)	72 (39.8)	0.004*
	30–39	25 (86.2)	4 (13.8)	
	≥ 40	3 (37.5)	5 (62.5)	
Facility currently working	Hospital	68 (63.6)	39 (36.4)	0.943
	Health center	69 (62.2)	42 (37.8)	
Location of the institution	Urban	112 (67.5)	54 (32.5)	0.018
	Rural	25 (48.1)	27 (51.1)	
Ownership of the institution	Government only	120 (65.2)	64 (34.8)	0.004*
	Private only	11 (39.3)	17 (60.7)	
	Both government and private	6 (100)	0 (0)	
Profession	BSc nurse	61 (63.5)	35 (36.5)	0.004
	Health officer	31 (75.6)	10 (24.4)	
	BSc Midwifery	8 (38.1)	13 (61.9)	
	Medical Lab Technologist	13 (44.8)	16 (55.2)	
	Pharmacist	24 (77.4)	7 (22.6)	
Work experience (years)	1–5	91 (59.5)	62 (40.5)	0.064*
	6–10	30 (71.4)	12 (28.6)	
	11–15	14 (82.4)	3 (17.6)	
	≥ 16	2 (33.3)	4 (66.7)	
Have you attended trainings on antimicrobial resistance?	Yes	16 (76.2)	5 (23.8)	0.274
	No	121 (61.4)	76 (38.6)	
Have you ever used antimicrobial sensitivity test result for treating patients?	Yes	5 (71.4)	2 (28.6)	1.000*
	No	132 (62.6)	79 (37.4)	
Do you get up to date information on antimicrobial resistance?	Yes	69 (68.3)	32 (31.7)	0.158
	No	68 (58.1)	49 (41.9)	

* Fisher’s exact value

### Association of socio-demographic characteristics and professional profiles with responses to knowledge questions

From a total of 184 participants working in governmental institutions, 61.4% (113) correctly responded to knowledge questions while only 32.1% (9 out of 28) working in private only and 33.3% (2 out 6) working in both government and private institutions gave correct answers. This difference was found statistically significant (p = 0.005). Regarding the profession of the participants, 83.9% of pharmacists, 68.3% of health officers, 50% of nurses, 48.3% of Laboratory technologists and 38.1% of midwives gave correct responses to knowledge questions. The proportional differences of giving correct responses among the profession was statistically significant (p = 0.002). On the other hand, statistical differences were not observed (p > 0.05) between sex, age groups, type of facility currently working in, location of the institution, work experiences, attending of training on antimicrobial resistance and usage of antimicrobial sensitivity test results (Table 4).

**Table 4:** Association of socio-demographic characteristics and professional profiles with responses to knowledge questions, Dire Dawa, Ethiopia, October to November 2015

Variables	Categories	Response to knowledge questions		p value
		Correct N (%)	Incorrect N (%)	
Sex	Male	87 (59.6)	59 (40.4)	0.315
	Female	37 (51.4)	35 (48.6)	
Age group	20–29	99 (54.7)	82 (45.3)	0.051*
	30–39	22 (75.9)	7 (24.1)	
	≥ 40	3 (37.5)	5 (62.5)	
Facility currently working	Hospital	55 (51.4)	52 (48.6)	0.142
	Health center	69 (62.2)	42 (37.8)	
Location of the institution	Urban	91 (54.8)	75 (45.2)	0.348
	Rural	33 (63.5)	19 (36.5)	
Ownership of the institution	Government only	113 (61.4)	71 (38.6)	0.005*
	Private only	9 (32.1)	19 (67.9)	
	Both government and private	2 (33.3)	4 (66.7)	
Profession	BSc nurse	48 (50)	48 (50)	0.002
	Health officer	28 (68.3)	13 (31.7)	
	BSc Midwifery	8 (38.1)	13 (61.9)	
	Medical Lab Technologist	14 (48.3)	15 (51.7)	
	Pharmacist	26 (83.9)	5 (16.1)	
Work experience (years)	1–5	86 (56.2)	67 (43.8)	0.615*
	6–10	25 (59.5)	17 (40.5)	
	11–15	11 (64.7)	6 (35.3)	
	≥ 16	2 (33.3)	4 (66.7)	
Have you attended trainings on antimicrobial resistance?	Yes	11 (52.4)	10 (47.6)	0.837
	No	113 (57.4)	84 (42.6)	
Have you ever used antimicrobial sensitivity test result for treating patients?	Yes	5 (71.4)	2 (28.6)	0.701
	No	119 (56.4)	92 (43.6)	
Do you get up to date information on antimicrobial resistance?	Yes	60 (59.4)	41 (40.6)	0.574
	No	64 (54.7)	53 (45.3)	

* Fisher’s exact value

### Interventions needed to combat antimicrobial resistance

Nine possible intervention mechanisms of antimicrobial resistance were listed and participants were asked to indicate the usefulness of each strategy in combating antimicrobial resistance. In general, more than 80% of the respondents had positive attitudes towards the strategies (considering very useful and useful responses as positive attitudes and not useful as negative attitude). The interventions considered very useful by the large number of respondents were updating about local antibiotic resistance patterns, 159 (72.9%), establish infection control committee, 155 (71.1%) and establish microbiology diagnostic services, 149 (68.3%) (Table 5). Regarding attitudes of respondents towards the intervention methods, statistically significant differences were observed among many variables, p < 0.05 (Table 8).

**Table 5:** Attitudes of health professionals towards interventions of antimicrobial resistance, Dire Dawa, Ethiopia, October to November 2015

Possible interventions	Responses							
	Very useful		Useful		Not useful		Not sure	
	N	%	N	%	N	%	N	%
Reduction of antibiotic use for outpatient setting	45	20.6	135	61.9	29	13.3	9	4.1
Access to current antibiogram	81	37.2	115	52.8	5	2.3	17	7.8
Antimicrobial usage policy	141	64.7	66	30.3	3	1.4	8	3.7
Updating about local antibiotic resistance patterns	159	72.9	41	18.8	3	1.4	15	6.9
Establish national antimicrobial resistance surveillance	140	64.2	71	32.6	0	0	7	3.2
Establish hospital infection control committee	155	71.1	63	28.9	0	0	0	0
Develop institutional guideline for antimicrobial use	146	67	65	29.8	7	3.2	0	0
Education on antimicrobial therapy for prescribers	148	67.9	59	27.1	11	5	0	0
Establish microbiology diagnostic services	149	68.3	66	30.3	0	0	3	1.4

### Magnitude of antimicrobial resistance problem

Respondents were asked about the importance of antimicrobial resistance in their institutions, in the country and in the globe. Regarding the severity of the problem in their own institution, 22.2% strongly agreed, 47.2% agreed, 15.1% disagreed and 17% did not know the status of the problem in their setting. Of the participants, 53.7, 42.2, 1.4, and 1.8% strongly agreed, agreed, disagreed and strongly disagreed, respectively, on the severity of the problem in their country, Ethiopia. As the situation is a worldwide problem, 40.8% strongly agreed, 50% agreed and 6.9% disagreed (Table 6). Concerning the magnitude of antimicrobial resistance problem in the participants’ institutions, statistically significant differences were noted between sex (p = 0.020), age group (p = 0.036) and access to up to date information (p = 0.039) (Tables 7, 8).

**Table 6:** Attitudes of health professionals towards the importance of antimicrobial resistance problem, Dire Dawa, Ethiopia, October to November 2015

Statements on antimicrobial resistance problem	Responses									
	St agree		Agree		Disagree		Str disagree		Don’t know	
	N	%	N	%	N	%	N	%	N	%
Antimicrobial resistance is a worldwide problem	89	40.8	109	50	15	6.9	3	1.4	2	0.9
Antimicrobial resistance is a problem in Ethiopia	117	53.7	92	42.2	3	1.4	4	1.8	2	0.9
Antimicrobial resistance is a problem in your institution	44	20.2	103	47.2	33	15.1	1	0.5	37	17

**Table 7:** Association of socio-demographic characteristics with attitude statements towards importance of antimicrobial resistance problem and causes of unnecessary antibiotic prescriptions, Dire Dawa, Ethiopia, October to November 2015

	Response categories					p value							
	Strongly agree	Agree	Disagree	Strongly disagree	Do not know	Sex	Age group	Facility	Location	Ownership	Experience	Training	Up to date information
Attitude statements towards the importance of antimicrobial resistance problem													
Antimicrobial resistance is a worldwide problem.	89 (40.8)	109 (50)	15 (6.9)	3 (1.4)	2 (0.9)	0.034*	0.412*	0.612*	0.020*	0.930*	0.001*	0.707*	0.357*
Antimicrobial resistance is a problem in Ethiopia.	117 (53.7)	92 (42.2)	3 (1.4)	4 (1.8)	2 (0.9)	0.000*	1.000*	0.030*	0.005*	0.002*	0.503*	0.928*	0.093*
Antimicrobial resistance is a problem in your institution.	44 (20.2)	103 (47.2)	33 (15.1)	1 (0.5)	37 (17)	0.020*	0.036*	0.888*	0.665*	0.093*	0.058*	0.000*	0.039*
Attitude statements towards causes of unnecessary antibiotic prescriptions													
Unnecessary antibiotic prescriptions are by patient push	35 (16.1)	81 (37.2)	54 (24.8)	42 (19.3)	6 (2.8)	0.000*	0.001*	0.516*	0.004*	0.040*	–	0.001*	0.555*
Unnecessary antibiotic prescriptions are due to treatment failure	43 (19.7)	98 (45)	60 (27.5)	13 (6)	4 (1.8)	0.626*	0.054*	0.858*	0.003*	0.037*	0.391*	0.053*	0.126
Unnecessary antibiotic prescriptions are for critically ill or immune-compromised patient	47 (21.6)	57 (26.1)	94 (43.1)	16 (7.3)	4 (1.8)	0.016*	0.464*	0.399*	0.844*	0.005*	0.732*	0.197*	0.058*
Unnecessary antibiotic prescriptions are for profit of hospitals/health centers	23 (10.6)	58 (26.6)	64 (29.4)	66 (30.3)	7 (3.2)	0.999*	0.077*	0.973*	0.590*	0.649*	0.537*	0.053*	0.002*

* Fisher’s exact value

**Table 8:** Association of socio-demographic characteristics with attitude statements towards interventions of antimicrobial resistance, Dire Dawa, Ethiopia, October to November 2015

Statements	Response categories				p value							
	Very useful	Useful	Not useful	Not sure	Sex	Age group	Facility	Location	Ownership	Experience	Training	Up to date information
Reduction of antibiotic use for outpatient setting	45 (20.6)	135 (61.9)	29 (13.3)	9 (4.1)	0.007*	0.000*	0.403*	0.065*	0.114*	0.000*	0.728*	0.103*
Access to current antibiogram	81 (37.2)	115 (52.8)	5 (2.3)	17 (7.8)	0.172*	0.054*	0.026*	0.483*	0.073*	0.322*	0.492*	0.016*
Antimicrobial usage policy	141 (64.7)	66 (30.3)	3 (1.4)	8 (3.7)	0.000*	0.031*	0.353*	0.138*	0.001*	0.193*	0.526*	0.002*
Updating about local antibiotic resistance patterns	159 (72.9)	41 (18.8)	3 (1.4)	15 (6.9)	0.834*	0.211*	0.043*	0.060*	0.255*	0.287*	0.529*	0.185*
Establish national antimicrobial resistance surveillance	140 (64.2)	71 (32.6)	–	7 (3.2)	0.000*	0.687*	0.012*	0.000*	0.056*	0.075*	0.101*	0.007*
Establish hospital infection control committee	155 (71.1)	63 (28.9)	–	–	0.293	0.296*	1.000	0.223	0.834*	0.399*	0.773	0.021
Develop institutional guideline for antimicrobial use	146 (67)	65 (29.8)	7 (3.2)	–	0.427*	0.782*	0.038*	0.075*	0.001*	0.605*	0.252*	0.189*
Education on antimicrobial therapy for prescribers	148 (67.9)	59 (27.1)	11 (5.0)	–	0.287*	0.095*	0.007	0.324*	0.000*	0.018*	0.843*	0.039
Establish microbiology diagnostic services	149 (68.3)	66 (30.3)	3 (1.4)	–	0.244*	0.225*	0.003*	0.437*	0.000*	0.020*	0.720*	0.049*

* Fisher’s exact value

### Causes of unnecessary antibiotic prescriptions

Participants were asked four questions on the causes of unnecessary antibiotic prescriptions such as patient push, treatment failure, critically ill/immunocompromised patient and prescription for institutional benefit. The belief of the participants regarding unnecessary prescriptions by patient push was 16.1% strongly agree, 37.2% agree, 24.8% disagree, 19.3% strongly disagree, and only 2.8% did not know. A large proportion of the participants, 45% agreed that unnecessary antibiotics prescriptions were due to treatment failures. 43.1% of the respondents disagreed that critically ill/immune-compromised patients cause unnecessary antibiotic prescriptions. On the question that unnecessary antibiotic prescriptions were for the benefit of the institution, the majority, 30.3%, of the participants were strongly disagreed (Table 9).

**Table 9:** Health professionals’ attitudes on causes of unnecessary antibiotic prescriptions, Dire Dawa, Ethiopia, October to November 2015

Statements on causes of unnecessary antibiotic prescriptions	Responses									
	Strongly agree		Agree		Disagree		Strongly disagree		Don’t know	
	N	%	N	%	N	%	N	%	N	%
Unnecessary antibiotic prescriptions are by patient push	35	16.1	81	37.2	54	24.8	42	19.3	6	2.8
Unnecessary antibiotic prescriptions are due to treatment failure	43	19.7	98	45	60	27.5	13	6	4	1.8
Unnecessary antibiotic prescriptions are for critically ill or immune-compromised patient	47	21.6	57	26.1	94	43.1	16	7.3	4	1.8
Unnecessary antibiotic prescriptions are for profit of hospitals/health centers	23	10.6	58	26.6	64	29.4	66	30.3	7	3.2

### Antibiotics prescription practices

Antibiotics prescription practices of health workers were elaborated in Table 10. Out of the total study participants (N = 218), 134 (61.5%) were practicing antibiotic prescription; all the 41 health officers, 78 nurses and 15 Midwives. The majority, 103 (76.9%) of the participants responded that standard treatment guidelines were available in their institutions. However, only 15.7% of the prescribers responded they ‘always’ referred the standard treatment guideline while almost half of them, 49.3% referred ‘some times’. Regarding the frequency of reviewing antibiotics prescriptions with senior/colleague, 30.6% some times, 27.6% often and only 9% always consult their seniors/colleagues to decide on antibiotics prescriptions. Respectively, 28.4 and 4.5% communicated rarely and never with seniors/colleagues.

**Table 10:** Antimicrobial prescription practices of health workers, Dire Dawa, Ethiopia, October to November 2015

Statements	Categories	Frequency (N = 134)	%
Is there standard treatment guideline in your institution?	Yes	103	76.9
	No	31	23.1
How frequent do you refer standard treatment guideline to prescribe antibiotics?	Always	21	15.7
	Often	37	27.6
	Some times	66	49.3
	Rarely	5	3.7
	Never	5	3.7
How frequent do you review your decision to prescribe antibiotics with a senior or colleague?	Always	12	9.0
	Often	37	27.6
	Some times	41	30.6
	Rarely	38	28.4
	Never	6	4.5
Is it difficult to select the correct antibiotic?	Yes	38	28.4
	No	96	71.6
How often do you face difficulty to select the correct antibiotic?	Always	5	3.7
	Often	10	7.5
	Some times	56	41.8
	Rarely	50	37.3
	Never	13	9.7
During the last year, how many times have you received some teaching in your department on antibiotic prescription?	None	106	79.1
	Once	14	10.4
	twice	8	6.0
	≥ 3 times	6	4.5
During the last year, how many times have you received training courses on antibiotic prescription?	None	115	85.8
	Once	9	6.7
	twice	10	7.5
	≥ 3 times	0	0

Although the majority, 71.6% of the prescribers responded that it was not difficult to select the correct antibiotics, only 9.7% reported that they never faced the problem. It was reported, 41.8% some times, 3.7% always and 37.3% rarely faced the difficulty. Almost four-fifth, 79.1% of the participants did not receive any teaching related to antibiotics prescription in their departments or institutions. Less proportion of the participants, 10.4, 6 and 4.5% received some teachings on antibiotics prescription once, twice and three or more times, respectively. Questions were asked on the attendance of formal trainings on antibiotics prescription and the majority, 85.8% never attended trainings while only 14.2% took once or twice.

## Discussions

The continuing emergence, development and spread of pathogenic organisms that are resistant to antimicrobials are a cause of increasing concern. Antibiotic resistance is causing not only increased morbidity and mortality but also a high economic burden, adding a load to health systems in low income countries [3, 4]. In Ethiopia, the effectiveness of currently available antimicrobial drugs is decreasing due to the increasing number of resistant strains causing infections so that available therapeutic options for such organisms are severely limited; the overall multidrug resistance among bacterial isolates was 59–79% [5, 6].

The control of antimicrobial resistance requires knowledge of factors causing antimicrobial resistance, good attitudes towards the intervention strategies as well as changes in antibiotic prescribing behavior of health workers. Health professionals are key stakeholders in prevention and control of antimicrobial resistance through appropriate prescription of antimicrobials, controlling transmission of drug resistant bacteria and promoting awareness in the community. Thus, this study came up with the assessment results of knowledge and attitudes of paramedical staffs towards antimicrobial resistance and their antibiotic prescription practices in Dire Dawa Administration, Ethiopia.

The current study showed that 62.8% of paramedical staffs had good knowledge on the factors causing antimicrobial resistance. This is similar to the study which has also reported a good knowledge among paramedical staffs [13]. This result is also comparable with the previous report that 63.5% of nurses had good knowledge on antimicrobial resistance [12]. These findings are hopeful as better knowledge may be correlated well with better health practices.

Level of knowledge of the study participants towards contributing factors to antimicrobial resistance was found statistically significant (p < 0.05) between sex, age groups, location of the institution (rural, urban), ownership of institution and profession. Males were found more knowledgeable (67.8% versus 52.8%, p = 0.044). However, there were no significant gender differences with respect to knowledge as reported from a cross sectional survey done in India [13]. This discrepancy may be due to small sample size in the current study.

Participants in the middle age group (30–39 years) were found more knowledgeable (86.2%) when compared with those in the lower (20–29 years, 60.2%) and higher (≥ 40 years old, 37.5%) age groups which was statistically significant, p = 0.004. This is different from the report from India that younger age subjects had better knowledge [13].

In this study, a statistically significant difference (p = 0.004) was observed in the level of knowledge among participants working in government and private health sectors (65.2% versus 39.3%). Practitioners in the urban facilities were observed giving more correct answers to knowledge questions as compared with rural practitioners (67.5% versus 48.1%) with statistically significant difference (p = 0.018). This difference may be due to the fact that urban practitioners have better access to web based information on antimicrobial resistance.

Based on the results of this survey, 77.4% of the participating pharmacists, 75.6% of Health officers, 63.5% of nurses, 44.8% of Lab technologists and 38.1% of Midwives had good knowledge regarding causes of antimicrobial resistance and these differences in proportion was statistically significant (p = 0.004). Other studies reported that differences in the level of knowledge were observed between professionals; nursing staffs and pharmacists had better knowledge about antibiotic effectiveness compared to other paramedical staffs [13]. Similarly, statistically significant difference was observed between physicians and nurses in some aspects of knowledge and beliefs on antimicrobial resistance as previously reported from Ethiopia [12].

Almost 90% of the interviewed health workers had not taken any training on antimicrobial resistance. Of the trained participants, 76.2% had good knowledge while 61.4% of non-trained had good knowledge on questions asked about factors causing antimicrobial resistance. However, the proportional difference in the level of knowledge among trained and non-trained respondents was not statistical significant, p = 0.274. Other study showed that those who had medical training had better knowledge [13]. It was also reported that 65% of physicians and 98% of nurses need further training on stewardship of antimicrobial resistance [12].

Higher proportion of study participants who had up to date information from various sources observed having of good knowledge as compared with those lacking access to source of information (68.3% vs. 58.1%) though the difference was not statistically significant (p = 0.158).

In the current study, participants responded that the most common causes of antimicrobial resistance were: patient poor adherence (96.5%), self prescription (95%), and empiric choice of antibiotics (94.5%). It was also reported by 92.7% of the participants that each widespread/overuse of antibiotics, inappropriate duration of antibiotic courses and poor infection control contributed for antimicrobial resistance. Similarly, it was reported that the most important perceived factors contributing for antimicrobial resistance development were patients’ poor adherence to prescribed antibiotics and widespread or overuse of antibiotics [12]. Likewise, a study conducted in Scotland, France and Spain stated that too many antibiotic prescriptions and inappropriate duration of antibiotic treatments were the leading factors [14, 15].

On the other hand, less proportion, almost 60%, of the study participants perceived as the use of broad spectrum antibiotics and promotion by pharmaceutical representatives promote antimicrobial resistance. Use of broad spectrum antibiotics was not well recognized though poor hand washing was well considered as a cause of antimicrobial resistance in the current study unlike other studies [12, 14, 15]. Hence, awareness on the usage of broad spectrum antibiotics should be created. Similarly, a cross sectional study from Sudan reported that respondents were less likely to perceive that pharmaceutical companies’ promotion of antibiotics as very important factors [16].

Regarding the intervention mechanisms of antimicrobial resistance, more than 80% of the respondents had positive attitudes towards the strategies (considering very useful and useful responses as positive attitudes and not useful as negative attitude) so as to combat the occurrence of antimicrobial resistance. The majority of the participants believed that updating local antibiotic resistance patterns (72.9%), establishing infection control committee (71.1%), and establishing microbiology diagnostic services (68.3%) were very useful intervention strategies to reduce antimicrobial resistance. These findings are more or less in line with the previous reports [12, 14, 16, 17]. This indicates that strengthening and incorporating these intervention measures into the healthcare system can reduce the occurrences of antimicrobial resistance.

Regarding the severity of antimicrobial resistance problem in their own institution, 22.2% strongly agreed, 47.2% agreed, 15.1% disagreed and 17% did not know the status of the problem in their setting. Compared with the perceived spectrum of problem by respondents in the world and nation wise, few participants recognized antimicrobial resistance as a problem in their local institutions. These findings are consistent with a study conducted in Ethiopia [12], Sudan [16], India [18] and DR Congo [19]. The lack of awareness on the magnitude of antimicrobial resistance problem in their institutions may be due to the scarcity bacteriological culture and susceptibility testing to assess the pattern of microbial resistances. The perception of participants towards the magnitude of antimicrobial resistance problem in their institutions was noted statistically significant between sex (p = 0.020), age group (p = 0.036) and access to up to date information (p = 0.039).

Concerning the causes of unnecessary antibiotic prescriptions such as patient push, treatment failure, critically ill/immunocompromised patient and institutional benefit, participants were asked to rate their beliefs. Based on the rating, strongly agree or agree, the most perceived driving forces were treatment failure (67.7%) and patient push (53.3%) followed by critically ill/immunocompromised patient (47.1%). These findings are in agreement with the previous report [12]. In the recent study, as antibiotic prescriptions were for the benefit of the institution was mentioned by only 37.2% of the participants. This shows the majority of the participants in the study area perceived that health sectors are non-profitable organizations that give health care services for the surrounding communities.

Antibiotics are among the most commonly prescribed drugs used in human medicine. However, up to 50% of all the antibiotics prescribed for people are not needed or are not prescribed appropriately. A serious diarrheal infection usually associated with antibiotic use causes nearly 250,000 hospitalizations and at least 14,000 deaths every year in the United States [20].

Referring the standard treatment guideline is very important for selecting the appropriate medications so as to provide quality health services thereby preventing the spread of drug resistant microorganisms. In the current study, the majority, 76.9% of the prescribers mentioned that standard treatment guidelines were available in their institutions. However, only 15.7% of the prescribers responded they ‘always’ referred the standard treatment guidelines and 28.4% rarely consult their seniors/colleagues to decide on antibiotics prescriptions. Therefore, the medical education strategies should aim, not only to increase the knowledge, but also to change the behavior and practices among medical students. Apart from teaching about antibiotic prescribing, the principles of the protocol development for antibiotic use in health care facilities should form an integral part of the health care system.

In regard to selecting the correct antibiotics for prescribing to the patients, 71.6% of the prescribers responded that it was not difficult though only few, 9.7% reported that they never faced the problem. The clinical effectiveness of antibiotics depends partially on their correct use, depending on patients, physicians and retailers [21]. This needs that prescribers should refer the available guidelines and communicate with their senior coworkers to select the appropriate antibiotics. This is because senior staff was the most common source of information about the antibiotics [22].

Almost four-fifth, 79.1% of the participants did not receive any teaching related to antibiotics prescription in their departments or institutions. Furthermore, the majority, 85.8% of the participants never attended formal trainings on antibiotics prescriptions. Learning about the antimicrobial prescribing in pharmacology must be connected clearly with the infection control in microbiology [23]. Furthermore, refreshment courses and short term trainings considerably change the patterns of antibiotics prescription practices, which needs to be incorporated into the capacity building of health care providers of all levels. Previous study reported that 91% of all physicians would like to have refresher courses on antibiotics [24].

## Limitation of this study

The strength of this study, in fact, is that it addresses the knowledge and attitude of paramedical staffs towards antimicrobial resistance as well as some aspects of their prescription practices among whom much research has not been conducted in the study area so far. This segment of population remains to be untouched due the fact that there might be a general perception that health professionals would have better understanding about antibiotics. As with most surveys, our study does have certain limitations; it is possible that the study participants might have given socially desirable answers rather than their true opinions or practices.

## Conclusions

This study generated important information on knowledge and attitude of paramedical staffs about antimicrobial resistance, and some aspects of their antibiotics prescription practices. It can be concluded that more than half of the respondents had good knowledge on the causes of antimicrobial resistance though considerable number of participants had poor knowledge according to the survey results. Almost 90% of the health workers had not taken any training on antimicrobial resistance. It was reported that the most common causes of antimicrobial resistances were patient poor adherence, self prescriptions and empiric choice of antibiotics.

Majority of the respondents had positive attitudes towards the intervention mechanisms suggesting that updating local antibiotic resistance patterns, establishing of infection control committee and microbiology diagnostic services were very useful strategies so as to combat the occurrences of antimicrobial resistances. In the majority of health institutions, standard treatment guidelines were available though a few referred the guideline for prescribing antibiotics. Furthermore, this survey indicated that four-fifth of the prescribers had never attended any training related to antibiotics prescription.

The results of this study identified areas of misconceptions and specific groups to be targeted for educational interventions regarding antimicrobial resistance. It is, therefore, suggested that a well-planned, organized and structured training programs should be undertaken to improve the appropriate use of antibiotics.

## Abbreviations

CDC: Centers for Disease Control and Prevention
SPSS: Statistical Package for Social Sciences
WHO: World Health Organization
